# Public Perception towards the COVID-19 Vaccine in Riyadh, Saudi Arabia

**DOI:** 10.3390/vaccines10060867

**Published:** 2022-05-28

**Authors:** Zeyad Kurdee, Samia Al-Shouli, Nouf AlAfaleq, Sultan Ayoub Meo, Alanoud Alshahrani, Aseel Alshehri, Noura Alkathiri, Shaden Bin Saiedan, Yara Alzahrani

**Affiliations:** 1 Department of Pathology, College of Medicine, King Saud University, Riyadh 11461, Saudi Arabia; zkordee@ksu.edu.sa; 2 Department of Pathology, Immunology Unit, College of Medicine, King Saud University, Riyadh 11451, Saudi Arabia; salshouli@ksu.edu.sa; 3 Department of Biochemistry, College of Science, King Saud University, Riyadh 11451, Saudi Arabia; nalafaleg@ksu.edu.sa; 4 Department of Physiology, College of Medicine, King Saud University, Riyadh 11461, Saudi Arabia; 5 College of Medicine, King Saud University, Riyadh 11451, Saudi Arabia; 439200250@student.ksu.edu.sa (A.A.); 439200230@student.ksu.edu.sa (A.A.); 439200411@student.ksu.edu.sa (N.A.); 439200105@student.ksu.edu.sa (S.B.S.); 439200173@student.ksu.edu.sa (Y.A.)

**Keywords:** COVID-19 pandemic, vaccine, public perception, acceptance rate, Saudi Arabia

## Abstract

The vaccination campaign against COVID-19 is an essential public health strategy to reach herd immunity, eradicate diseases, and prevent a pandemic. This study aimed to investigate the acceptance rate of the COVID-19 vaccine among people in Riyadh, Saudi Arabia. This cross-sectional study was conducted in the Department of Pathology, College of Medicine, King Saud University, Riyadh, Saudi Arabia. Out of the 922 participants involved, 294 (31.9%) were male and 628 (68.1%) were female, with a mean age of 30–49 years. A bilingual, self-administered, computer-based questionnaire was designed and distributed through social media platforms. In total, 900 participants (97.6%) showed a high acceptance rate of the vaccine. The vaccine acceptance rate was higher among people aged 60 years and above than in other age groups (*p* = 0.008) and single individuals compared to other groups (*p* = 0.003). The results reveal a relatively high acceptance level of the COVID-19 vaccine among study participants. Importantly, regression analysis results show that female gender and elderly participants are more likely to accept the COVID-19 vaccine than their counterparts. Moreover, the main factor that influenced the participants’ perception of the COVID-19 vaccine was the proper timely scientific recommendations.

## 1. Introduction

The COVID-19 pandemic has made a significant impact on the public health system, the economy, and the psychological well-being of the population in several parts of the world, leading to the tragic loss of millions of human lives worldwide [1,2,3]. Several nations have implemented decisive measures to mitigate the spread of COVID-19 infection and promote positive clinical and socioeconomic outcomes, including the imposition of national or local lockdowns, the mandatory wearing of face masks, social distancing in public places, and quarantine for those infected or who have come in contact with infected patients [4,5]. Vaccination is a gold standard for inducing immunity and preventing diseases. Vaccines are one of the most convenient care measures available to improve health outcomes and quality of life, and make healthy life expectancy more equitable by preventing and controlling infectious diseases [6].

Vaccine hesitancy, described as a “delay in acceptance or refusal of vaccination despite the availability of vaccination services”, poses a potential threat to global public health [7]. Several studies have reported that vaccine hesitancy is influenced by factors such as misconceptions about the need for vaccination, doubts about the credibility of pharmaceutical companies, lack of vaccine and disease literacy, lack of confidence in vaccine safety and efficacy, side effects of the vaccine, and whether the vaccine is provided for free by the government [8,9].

A global overview about flattening the curve of COVID-19 vaccine rejection provided evidence that vaccination hesitancy was high even in countries severely affected by the pandemic. It is suggested that herd immunity can be achieved when governments and successful social campaigners implement a wide range of support to start mandatory preventative vaccination programs combined with coordinated efforts in education [10]. Another study showed that vaccine acceptance is based on education, employment, and ethnicity [11,12]. The health officials introduced strategies to improve vaccine acceptance among their populations by highlighting the increasing perceived risk for COVID-19 in communities [13].

In Saudi Arabia, a recent study revealed high variability in people’s willingness to accept the COVID-19 vaccination among different socio-demographic determinants. The willingness to accept the future COVID-19 vaccine was relatively high among older age groups. It was also high among highly educated married participants, non-Saudi participants, and those employed in the government sectors [14]. Moreover, another study demonstrated a low acceptance rate, showing that while 52% of the Saudi population was uncertain or did not report any intention to take the vaccine, 48% were willing to receive the vaccine. A few factors are associated with COVID-19 vaccination intentions in Saudi Arabia, such as the perceived risk factors, past vaccination behavior, previous contraction of COVID-19, and support for compulsory vaccination [15]. Literature that evaluates the prevalence of the COVID-19 vaccine and the factors that influenced its acceptance after the COVID-19 vaccination campaign started in Saudi Arabia in January 2021 [16] does exist, but is lacking from various parts of the region. Therefore, this study aimed to investigate the acceptance rate of the COVID-19 vaccine among people in Riyadh, Saudi Arabia. Moreover, this study also sought to compare the acceptance rate of the COVID-19 vaccine among people with and without chronic diseases.

## 2. Subjects and Methods

Study design and settings: A cross-sectional study was conducted between June 2021 and December 2021 on the residents of Riyadh City (both Saudi and non-Saudi) citizens. A bilingual (Arabic and English), self-administered, computer-based questionnaire was designed and distributed via a link survey to various social media platforms and other mailing platforms, including email and WhatsApp. Participants were asked to share the survey with others.

Sample size: The study participants were Saudi and non-Saudi, male and female, residents in Riyadh city, and aged 18 years and above; for this study, a sample size of 922 participants was required based on a 50% population proportion, 95% confidence interval, and 5% margin of error, calculated using the formula *n* = Z² × *p*(1 − *p*)/d². The results were multiplied by 2 and added a potential 20% non-response. Our inclusion criteria were that participants needed to be at least 18 years old and above, live in Riyadh City, and included both people who did and did not receive the COVID-19 vaccine. Moreover, we included people who did have chronic diseases and people who did not have any chronic conditions. We excluded participants below 18 or who live in a city other than Riyadh.

The study variables: The study variables were socio-demographic characteristics (marital status, age, and gender), education levels, nationality, history of chronic conditions, a previous recipient of the flu vaccine, and if they had a family member who had previously contracted COVID-19. The outcome variables were acceptance of the COVID-19 vaccine, approval of the COVID-19 vaccine between chronically ill people, and knowledge of the COVID-19 vaccine.

Questionnaire development: The questionnaire was designed by project members. It was initially distributed to a pilot sample consisting of 15 participants to check for any technical misunderstandings or contraindications. The survey was made as short in length as possible. It was clarified that the information would be used only for medical research purposes, as the participants were not identified and had the right to withdraw at any stage.

Data analysis: The data were analyzed using SPSS statistical software, version 24.0. Simple descriptive statistics (mean, standard deviation, frequencies, and percentage) were used to describe the quantitative and categorical variables: the socio-demographic characteristics, knowledge about the COVID-19 vaccine, and vaccine acceptance rate. Pearson’s chi-square test was used to assess the association between the categorical variables. The Student t-test was used to compare the mean values; logistic regression analysis was used with acceptance of the COVID-19 vaccine as a dependent variable.

Ethical consideration: This research was conducted after the approval of the Institutional Review Board, King Saud University, College of Medicine, and informed consent was obtained from the participants at the beginning of the questionnaire.

## 3. Results

Out of 922 participants, 294 (31.9%) were male and 628 (68.1%) were female. The majority of the participants were Saudi Arabian nationals (96.1%). The other socio-demographic characteristics are shown in (Table 1).

Regarding previous infection with COVID-19 and having chronic diseases, we found that most of the participants, 709 (76.9%), had not been infected, and 383 (41.5%) had a family member that had been infected. When asked about any ongoing chronic diseases, we found that about a third of the 270 (29.3%) participants suffered from a chronic illness, with 90 (33.3%) of them reporting to have hypertension and 87 (32.2%) having diabetes mellitus. However, 44.8% of the participants reported that having chronic diseases did not affect their decision to receive the COVID-19 vaccine. The same proportion reported that it increased their desire to receive the vaccine, with only 8.5% reporting that it decreased their willingness to take up the vaccine. When participants were asked if they had received the COVID-19 vaccine, we found that 81% had received two doses while 13% had received two doses with natural immunity after recovering from COVID-19 infection.

Regarding knowledge about the COVID-19 vaccine, 63.4% of the participants agreed that they had received adequate information from the Ministry of Health. Concerning the vaccine’s side effects, the majority (73.5%) reported having a fever and experiencing pain at the needle site (68.4%). The factors that affected the participants’ opinion about the COVID-19 vaccine were scientific recommendations (43.8%), followed by family (29%), social media (14.9%), friends (3.9%), and other factors such as work and information from the Saudi Ministry of Health. Most of the participants (86.6%) agreed that the main factor that encouraged them to receive the vaccine was to protect themselves and the health of the community, and 85.1% of the participants agreed that the multiplicity of vaccination centers and the ease of the procedures also encouraged them to receive the vaccine. In comparison, 42% agreed that the multiplicity of companies producing the vaccine was a factor that inspired them. When the participants were asked about taking the seasonal flu vaccine before the COVID-19 pandemic, 60% reported that they had not (Table 2).

For factors associated with the acceptance rate of the COVID-19 vaccine, we conducted a chi-square test to explore the relationship between different socio-demographic characteristics and the history of chronic disease concerning the approval of the COVID-19 vaccine. A statistically significant association (*p* < 0.05) was found with the following factors: age and marital status. Our study results indicate that the highest percentage of acceptance for age groups was among participants aged 60 years or above when compared to other age groups (*p* = 0.008). We also recorded a higher level of acceptance in single individuals when reached in other groups (*p* = 0.003) (Table 3).

We conducted a logistic regression test with acceptance of the COVID-19 vaccine as the dependent variable. The results show that the acceptance rate of the COVID-19 vaccine was decreased among married people (odds ratio = 0.087, *p*-value = 0.012) (Table 4). Participants were asked if chronic disease affected their decision to receive the COVID-19 vaccine; 44.8% said that it did not affect their decision, 44.8% said that their desire to receive the vaccine increased, and only 8.5% said that their desire decreased.

## 4. Discussion

The present study evaluates the acceptance rate of the COVID-19 vaccine using the general population in Riyadh, Saudi Arabia. The results reveal that 97.6% of the study participants received the COVID-19 vaccine, and 2.4% did not take it. This indicates that most of the participants accepted the COVID-19 vaccine. Our study population acceptance rate is nearly in agreement with other studies conducted in China and the United States [17,18].

The Chinese research that compared the acceptance rate between health care workers (HCW) and the general population reported that 76.4% of 352 HCW and 72.5% of 189 members of the general population intended to take the COVID-19 vaccine [17]. In the United States, 80% of 3133 participants accepted the COVID-19 vaccine [18]. However, our acceptance rate is higher by 37.5% than that of a study conducted in Kuwait in which the acceptance rate was 53.1% [19]. This difference is attributed to multiple factors, such as a prior history of receiving the influenza vaccine and education level; all of these variables affect the acceptance rate in the Kuwait population [19]. In an Egyptian study, the authors found that 51.8% of the participants were concerned about the potential unexpected future effects of the vaccine [20]. Moreover, other studies in Saudi Arabia showed that 64% of the participants accepted the vaccine with no demographic factors associated with the acceptance rate [21].

The most significant factor associated with acceptance of the COVID-19 vaccine was age. Acceptability was highest among subjects aged 60 years and above (100%). Conversely, in a study conducted in Kuwait, the acceptance rate was higher among subjects in the lower age group of 21–24 years (74.3%) and lower among those aged 55–64 years (35.3%); other studies have shown that acceptability increases with age [19]. Moreover, there was no difference in the acceptance rate associated with other factors when we performed the statistical analysis.

It is good to have a high acceptance rate for the COVID-19 vaccine (90.6%), which is sufficient to generate herd immunity. The increased acceptance rate in Riyadh is due to multiple factors, as most of the population (63.4%) received adequate information about the COVID-19 vaccine from the Ministry of Health. The population receiving enough information from the right sources is an essential factor contributing to a high acceptance rate. Moreover, the multiplicity of vaccination centers and the ease of the procedures played a role in the increased acceptance rate. In addition, when participants were asked about other factors that encouraged them to receive the COVID-19 vaccine, 86.6% agreed that protecting their health and the health of their communities was a motivational factor that contributed to them receiving the vaccine; this was followed by the opportunity to return to the workplace and schools (79.7%).

Positively, we found that people with chronic diseases are more inclined towards acceptance of the COVID-19 vaccine than people without any chronic conditions. Patients with COVID-19 infection who also have a pre-existing cardiovascular disease, hypertension, diabetes mellitus, congestive heart failure, chronic kidney disease, and cancer have a greater risk of death from COVID-19 [22]. Conversely, a study conducted in Malaysia found that people with chronic conditions have lower acceptance rates than people who do not have any chronic diseases. Early vaccination for this population is essential to protect their health and reduce the risk of death. Therefore, there is a need to prioritize vaccinating this population and extend awareness about the risk of COVID-19 and the importance of vaccination [23].

## 5. Conclusions

The study results reveal a relatively high acceptance level of the COVID-19 vaccine among participants. Importantly, regression analysis showed that female and elderly (60 years or above) participants were more likely to accept the COVID-19 vaccine than their counterparts. Moreover, the main factor that affected the participants’ opinion about the COVID-19 vaccine was scientific recommendations. Health authorities may implement strategies that provide trusted information to increase awareness about the vaccines’ importance, safety, and efficacy.

## Figures and Tables

**Table 1:** Socio-demographic characteristics of the study participants (n = 922).

Socio-Demographic Characteristics		n (%)
Gender	Male	294 (31.9)
	Female	628 (68.1)
Age (years)	18–29	345 (37.4)
	30–49	349 (37.9)
	50–59	198 (21.5)
	60 or above	30 (3.3)
Nationality	Saudi	886 (96.1)
	Non-Saudi	36 (3.9)
Marital status	Single	365 (39.6)
	Married	557 (60.4)
Level of education	High school or below	187 (20.3)
	Diploma degree	89 (9.7)
	Bachelor’s degree	555 (60.4)
	Postgraduate degree	91 (9.9)

n = Numbers.

**Table 2:** Participants’ responses toward knowledge about the COVID-19 vaccine and its affecting factors (n = 922).

Question	Agree n (%)	Partially Agree n (%)	Disagree n (%)	I Don’t Know n (%)
Is the amount of information you got about the COVID-19 vaccine from the Ministry of Health sufficient?	585 (63.4)	206 (22.3)	77 (8.4)	54 (5.9)
To your Knowledge, does the COVID-19 Vaccine affect fertility?	34 (3.7)	43 (4.7)	369 (40)	476 (51.6)
The multiplicity of places and ease of procedures encourage you to receive the COVID-19 Vaccine?	785 (85.1)	78 (8.5)	34 (3.7)	25 (2.7)
Does the multiplicity of companies producing the COVID-19 Vaccine affect your decision to get it?	387 (42)	257 (27.9)	192 (20.8)	86 (9.3)
If the COVID-19 Vaccine becomes annual, would you get it?	353 (38.3)	170 (18.4)	225 (24.4)	174 (18.9)
Would you recommend the Vaccine to your friends and family?	733 (79.5)	81 (8.8)	52 (5.6)	56 (6.1)
	Yes	Occasionally	No	I don’t know
Before the COVID-19 pandemic, did you commit to annually having the seasonal flu vaccine?	181 (19.6)	175 (19)	553 (60)	13 (1.4)

n = numbers.

**Table 3:** Factors associated with the acceptance of the COVID-19 vaccine (n = 922).

Variable	Did You Receive the COVID-19 Vaccine?				
	Yes n (%)	No (%)	Odds Ratio	95% CI	*p*-Value
Age (years)					0.008
18–29	341 (98.8)	4 (1.2)	1		
30–49	342 (98)	7 (2)	0.57	0.17–1.98	
50–59	187 (94.4)	11 (5.6)	0.2	0.06–0.64	
60 or above	30 (100)	0 (0)	1.78	0	
Gender					0.648
Male	286 (97.3)	8 (2.7)	1		
Female	614 (97.8)	14 (2.2)	1.23	0.51–2.96	
Nationality					0.875
Saudi	865 (97.6)	21 (2.4)	1		
Non-Saudi	35 (97.2)	1 (2.8)	0.85	0.11–6.50	
Marital status					0.003
Single	363 (99.5)	2 (0.5)	1		
Married	537 (96.4)	20 (3.6)	0.15	0.03–0.64	
Education level					0.562
High school	181 (96.8)	6 (3.2)	1	0.23–3.89	
Diploma degree	86 (96.6)	3 (3.4)	0.95	0.65–5.04	
Bachelor degree	545 (98.2)	10 (1.8)	1.81	0.24–3.98	
Postgraduate degree	88 (96.7)	3 (3.3)	0.97	0.24–3.98	
Chronic disease					0.46
No	638 (97.9)	14 (2.1)	1		
Yes	262 (97)	8 (3)	0.72	0.30–1.73	

n = numbers.

**Table 4:** Logistic regression with acceptance toward COVID-19 vaccine as the dependent variable (n = 922).

Variable	Odds Ratio	95% CI for Odds Ratio		*p*-Value
		Lower	Upper	
Age (years)				
18–29	1			0.303
30–49	2.406	0.508	11.408	0.269
50–59	0.937	0.192	4.571	0.935
60 or above	84.167	0.000	.	0.998
Gender				
Male	1			0.963
Female	1.024	0.369	2.845	
Nationality				
Saudi	1			0.815
Non-Saudi	0.778	0.095	6.385	
Marital status				
Single	1			0.012
Married	0.087	0.013	0.586	
Education level				
High school	1			0.374
Diploma degree	1.654	0.373	7.326	0.508
Bachelor’s degree	2.626	0.877	7.863	0.085
Postgraduate degree	1.692	0.334	8.570	0.525
Chronic disease				
No	1			0.822
Yes	1.119	0.420	2.979	

## Data Availability

May be provided on reasonable request to the corresponding author.
